# *Amaranthus leucocarpus* lectin recognizes a moesin-like O-glycoprotein and costimulates murine CD3-activated CD4^+^ T cells

**DOI:** 10.1002/iid3.58

**Published:** 2015-06-05

**Authors:** Maria Arenas-Del Ángel, Martha Legorreta-Herrera, Guillermo Mendoza-Hernández, Yonathan Garfias, Raul Chávez, Edgar Zenteno, Ricardo Lascurain

**Affiliations:** 1 Departamento de Bioquimica, Facultad de MedicinaUniversidad Nacional Autónoma de México; 2 Laboratorio de Inmunologia Molecular, Facultad de Estudios Superiores ZaragozaUniversidad Nacional Autónoma de México; 3 Unidad de Investigación Instituto de Oftalmologia “Fundación Conde de Valenciana”; 4 Departamento de Investigación en BioquimicaInstituto Nacional de Enfermedades Respiratorias “Ismael Cosio Villegas”, México

**Keywords:** Lectin, lipid raft, moesin-like protein, O-glycosylation, oligosaccharide recognition

## Abstract

The Galβ1,3GalNAcα1,O-Ser/Thr specific lectin from *Amaranthus leucocarpus* (*ALL*) binds a ∼70 kDa glycoprotein on murine T cell surface. We show that in the absence of antigen presenting cells, murine CD4^+^ T cells activated by an anti-CD3 antibody plus *ALL* enhanced cell proliferation similar to those cells activated via CD3/CD28 at 48 h of culture. Moreover, *ALL* induced the production of IL-4, IL-10, TNF-alpha, and TGF-beta in CD3-activated cells. Proteomic assay using two-dimensional electrophoresis and far-Western blotting, *ALL* recognized two prominent proteins associated to the lipid raft microdomains in CD3/CD28-activated CD4^+^ T cells. By mass spectrometry, the peptide fragments from *ALL*-recognized proteins showed sequences with 33% homology to matricin (gi|347839 NCBInr) and 41% identity to an unnamed protein related to moesin (gi|74186081 NCBInr). Confocal microscopy analysis of CD3/CD28-activated CD4^+^ T cells confirmed that staining by *ALL* colocalized with anti-moesin FERM domain antibody along the plasma membrane and in the intercellular contact sites. Our findings suggest that a moesin-like O-glycoprotein is the *ALL*-recognized molecule in lipid rats, which induces costimulatory signals on CD4^+^ T cells.

## Introduction

Activation of CD4^+^ T cells is a crucial event in the adaptive immune response, since these cells control humoral and cellular immunity through the recognition of specific antigens 1. The activation process initially involves interaction between T cell receptor (TCR) and MHC-peptide complexes on antigen-presenting cells (APCs) resulting in the formation of an immunological synapse 1. Receptor–ligand complexes implicated in the immunological synapse are glycoproteins, which help both cell junction and lipid raft mobility on the membrane surface 2. Lipid rafts are membrane microdomains that concentrate signalling molecules required for cellular costimulation and activation 3 in which the band 4.1, ezrin, radixin, and moesin (FERM) family proteins have an essential role in organizing the interactions among transmembrane proteins and the underlying cytoskeleton 4.

T cell activation requires stimulation via TCR and costimulation signals; a powerful costimulatory pathway is provided through the CD28 molecule; however, other lipid raft-proteins have been shown to generate enough costimulation to activate T cells 5. Some studies have shown that the saccharide portion of glycosylated proteins, such as CD2 and CD8, have an important role in the co-receptor interactions as reviewed 6. For this reason, it is important to know how the saccharide structure influences many of these processes.

Cell surface oligosaccharides have been shown to play an important role in recognition events 6. Oligosaccharides may be cross-linked by lectins, which are proteins that recognize saccharides attached to glycosylated molecules in a highly specific manner 7. *Amaranthus leucocarpus* lectin (*ALL*) has been shown to have an affinity for N-Acetyl-D-Galactosamine (GalNAc) in either the Thomsen–Friedenreich antigen (Galβ1,3GalNAc α1,O-Ser/Thr) or Tn antigen (GalNAc α1,O-Ser/Thr) 8. This lectin has been isolated from *A*. *leucocarpus* seeds 9, and its hemagglutination activity is specifically inhibited by GalNAc 8–10. *ALL* binds murine thymocytes and thymic dendritic cells, peritoneal macrophages, and peripheral blood T cells, but not B cells 11–13. *ALL* recognizes a ∼70 kDa O-glycoprotein, which increases its expression on CD4^+^ T cells stimulated by concanavalin-A for 48 h 13. The isolation of the *ALL*-recognized O-glycoprotein from murine thymocytes showed that it has blocked N-terminal amino acid residue 14. Moreover, total cells from murine lymph node stimulated by an anti-CD3 antibody in the presence of *ALL*, showed an increase in the T cell proliferation 15. Here, we confirm the ability of *ALL* to induce proliferation of murine CD4^+^ T cells, which were stimulated by a suboptimal dose of anti-CD3 antibody in the absence of APCs along 48 h cell culture. Under these conditions, we show production of cytokine transcripts as well as intracellular and soluble cytokines, and the partial characterization of the main lipid raft glycoprotein recognized by *ALL*.

## Materials and Methods

### Lectin, reagents, and antibodies

*A. leucocarpus* seeds were obtained in Tulyehualco (Mexico) and the lectin was purified by affinity chromatography as described previously 9. *ALL* was labeled with the N-hydroxysuccinimide ester of biotin from Pierce Chemical (Rockford, IL) with a label/protein ratio of 2:1 16. Phycoerythrin (PE)-labeled rat anti-mouse CD4, biotin-labeled hamster anti-mouse CD3∊ chain (145-2C11) monoclonal antibodies (mAbs), and PE-labeled rat IgG2a, kappa mAb (used as isotype control); purified no azide/low endotoxin (NA/LE) hamster anti-CD3 (clone 145-2C11) or anti-CD28 (clone 37.51) mAbs (used to activate T cells); PE-cyanine (Cy) 5-, fluorescein isothiocyanate (FITC)-, and CyChrome (CyChr)-labeled streptavidin; FITC-labeled rat anti-mouse mAb to IL-10, and IFN-gamma, PE-labeled rat anti-mouse mAb to TNF, biotin-labeled rat anti-mouse mAb to IL-4, and IL-2; FITC-labeled rat IgG2b, FITC-, PE-labeled rat IgG1 (used as isotype controls), mouse Th1/Th2/Th17 cytokine kit, were purchased from BD Biosciences (San Diego, CA). Rabbit anti-mouse TGF-beta polyclonal Ab, FITC-labeled goat anti-rabbit IgG, rabbit anti-mouse moesin FERM domain (EP1863Y) mAb were acquired from Abcam (Cambridge, MA). Alexa Fluor 546-labeled donkey anti- rabbit IgG (H+L) antibody and HyClone foetal bovine serum were from Life Technologies (Thermo Fisher Scientific, Inc. Waltham, MA). The magnetic antibody cell sorting (MACS) kit for isolation of murine CD4^+^ cells was purchased from Miltenyi Biotec (Bergisch Gladbach, Germany). Sodium pyruvate, l-glutamine, and β-mercaptoethanol were from Gibco BRL (Rockville, MD). Horseradish peroxidase-labeled goat anti-mouse IgG polyclonal antibody was from R&D System, Inc. (Minneapolis, MN). Penicillin and streptomycin were from In Vitro Company (Mexico City, Mexico). Carboxyfluorescein succimidyl ester (CFSE) was from Invitrogen (Camarillo, CA). Vectashield (mounting medium with diamidino-2-phenylindole [DAPI] fluorescent dye) was purchased from Vector Laboratories, Inc. (Burlingame, CA). Mini RNeasy and Omniscript RT kits were from Qiagen (Carlsbad, CA). Ampli-Taq polymerase was from Applied Biosystems (Branchburg, NJ). The mini complete protease inhibitors kit was from Roche Diagnostics GMBH (Mannheim, Germany). Bovine serum albumin fraction V (BSA) ≥95% purity, RPMI-1640 culture medium, Coomassie brilliant blue R-250, trypan blue, Triton X-100 Ultra-pure, polyoxyethylenesorbitan monolaurate (Tween-20), dimethyl sulfoxide, methyl-β-cyclodextrin (MβCD), peroxidase-labeled extravidin, saponin, biotin-labeled cholera toxin B subunit, brefeldin-A from *Penicillum brefeldianum*, OptiPrep, ethylenediamine tetraacetic acid (EDTA), sodium azide, Trizma base, HEPES, and other salts were from Sigma–Aldrich (St. Louis, MO). DC protein assay kit (500-0116), ReadyPrep 2D cleanup kit and reagents for SDS–PAGE were from Bio-Rad Laboratories (Hercules, CA). Reagents for 2-D electrophoresis, strips, and Reswelling Tray were acquired from GE Healthcare (Fairfield, CT). All solutions were prepared with Milli-Q water (Millipore, Bedford, MA).

### Mice

Six- to eight-week-old male Balb/c mice were killed by cervical disruption in accordance to the guidelines accredited by the Institutional Research and Ethics Committee. Cells from the axial lymph nodes were obtained, washed, and suspended in RPMI-1640 medium culture. Cell viability was assessed by the trypan blue dye exclusion test.

### Purified CD4^+^ T cell culture and proliferation assays

CD4^+^ T cells were isolated from lymph nodes using a MACS negative selection kit according to the manufacturer's instructions (Miltenyi Biotec). Purity of CD4^+^ T cells was assessed by flow cytometry using PE-labeled anti-CD4 mAb and CyChr-streptavidin after biotin-labeled anti-CD3 mAb. The purity and viability percentages of cell suspensions were 95–98%.

Purified CD4^+^ T cells at a density 1 × 10^7^/mL of RPMI-1640 medium were stained with CFSE according to described method 17. In brief, 1 mL of suspended cells in RPMI medium, were incubated with 15 μL of 0.5 mM CFSE (prepared from a 5 mM stock solution dissolved in dimethyl sulfoxide) for 10 min at 37°C in darkness. After incubation, cells were washed twice in 8 mL of RPMI medium supplemented with 10% heat-inactivated fetal calf serum, 1% l-glutamine, 100 mM sodium pyruvate, 100 IU penicillin, 50 µg/mL gentamicin, 100 µg/mL streptomycin, and 50 mM β-mercaptoethanol (supplemented-RPMI medium), and cell viability was evaluated by trypan blue dye exclusion test. CFSE-treated cells were cultured in a 96-well plate (Nunc, Thermo Fisher Scientific, Inc. Waltham, MA) with a supplemented-RPMI medium and stimulated with either 0.1, 1, or 5 µg/mL of plate-immobilized anti-CD3 mAb alone or in the presence of 1 µg/mL soluble anti-CD28 mAb or *ALL* at different concentrations (5, 10, 15, or 20 μg/mL) during 24, 48, 72, and 96 h at 37°C in a 5% CO_2_ atmosphere. Cells cultured with anti-CD28 mAb plus *ALL* or with these reagents separately were used as controls. Both CFSE-untreated and non-stimulated cells were also used as controls. At the end of the culture periods, the cells were acquired on a FACSCalibur flow cytometer (BD Biosciences, San Jose, CA) and analyzed by the FlowJo software (Tree Star, Inc. Ashland, OR). To evaluate the cell divisions that have occurred under stimulation, the FlowJo proliferation platform was used. A histogram based on the fluorescence intensity of unstimulated CFSE-stained cells, which lay within a scatter gate, was performed to differentiate the divided cells of lower intensity. The subsequent gates enclosed cell populations with progressive twofold decreases in fluorescent intensity. Cell proliferation was assessed by percentage of divided cells, cell proliferation index, and division index. Additionally, images from activated cells were acquired on a Zeiss Axivert 25 inverted microscope (Carl Zeiss, Göttingen, Germany).

### Membrane cholesterol depletion by methyl-β-cyclodextrin

Purified CD4^+^ T cells were stimulated by a 1 µg/mL immobilized anti-CD3 mAb alone or in the presence of 1 µg/mL soluble anti-CD28 mAb or 5 µg/mL *ALL*, for 48 h of culture. Once cultured, cells (5 × 10^5^) were washed in phosphate-buffered saline (PBS) and incubated with 10 mM MβCD for 30 min at 37°C 18,19. Then, cells were washed in PBS containing 0.1% BSA and 0.1% sodium azide (PBS–BSA buffer), incubated with biotin-*ALL* (15 μg/mL) for 30 min at 4°C, followed by a second incubation with CyChr-streptavidin at dilution 1:400 and analyzed by flow cytometry. Non-activated cells incubated with CyChr-streptavidin after biotin-*ALL*, and activated-cells incubated only with CyChr-streptavidin were used as controls.

### Flow cytometry analysis

The percentage of cell surface molecules on purified CD4^+^ T cells was analyzed by direct or indirect immunofluorescence. In brief, cells were washed in a PBS–BSA buffer and stained with fluorescent reagents during 15 min at 4°C for incubation. Afterwards, cells were washed in PBS–BSA buffer, suspended in FACS-Flow buffer and acquired in the FACSCalibur flow cytometer (BD Biosciences). In each case, 25 × 10^3^ cells were counted in linear mode for side and forward scatter and by log amplification for fluorescent cells. Fluorochrome-labeled isotype-matched control mAbs or fluorescent-second reagents were used to evaluate background staining. Data from cytometer were analyzed by the FlowJo software (Tree Star, Inc.).

### Cytokine mRNA production

Purified CD4^+^ T cells (5 × 10^6^) were simultaneously activated by 1 µg/mL immobilized anti-CD3 mAb plus 1 µg/mL anti-CD28 mAb or 1 µg/mL immobilized anti-CD3 mAb plus 5 µg/mL *ALL* (optimal concentrations) for 48 h of culture. Non-stimulated cells were used as control. To evaluate cytokine transcripts, cells were washed in sterile PBS, and total RNA was isolated by using the mini RNeasy kit according to manufacturer's instructions (Qiagen) and quantified by a spectrophotometer at 285 nm. One microgram of an RNA sample was reverse-transcribed through the Omniscript RT kit and 1 μg from the resulting cDNA was used to amplify the IL-2, IL-4, IL-10, TNF-α, IFN-γ, and TGF-β genes by polymerase chain reaction (PCR). Each cDNA sample was amplified in duplicate as described 20; the sets of primers and the cDNA concentration were calibrated for a number of cycles to obtain amplicons in the linear phase of amplification. The following gene specific primer sequences were used: (IL-2) forward 5′ ATG TAC AGC ATG CAG CTC GCA TC 3′, reverse 5′ GGC TTG TTG AGA TGA TGC TTT GAC A 3′; (IL-4) forward 5′ ACA GGA GAA GGG CGC CAT 3′, reverse 5′ GAA GCC CTA CAG ACG AGC TCA 3′; (IL-10) forward 5′ ATG CAG GAC TTT AAG GGT TAC TTG GGT T 3′, reverse 5′ ATT TCG GAG AGA GGT ACA AAC GAG GTT GTT T 3′; (TNF-α) forward 5′ ATG AGC ACA GAA AGC ATG ATC CGC 3′, reverse 5′ CCA AAG TAG ACC TGC CCG GAC TC 3′; (IFN-γ) forward 5′ GAA AGC CTA GAA AGT CTG AAT AAC T 3′, reverse 5′ ATC AGC AGC GAC TCC TTT TCG GCT T 3′; (TGF-β) forward 5′ GAC CGC AAC AAC GCC ATC TA 3′, reverse 5′ GGC GTA TCA GTG GGG GTC AG 3′, and (β-actin) forward 5′ GTG GGC CGC TCT AGG CAC CAA 3′, reverse 5′ CTC TTT GAT GTC ACG CAC GAT TTC 3′. PCR reactions were performed in a total volume of 30 µL buffer containing 50 mM KCl, 10 mM Tris-HCl, pH 8.3; 0.1 mg/mL gelatin, 2 mM MgCl_2_, 100 nM of each primer, 200 mM dNTPs, and 0.5 U of ampli-Taq polymerase/75 ng of cDNA. After 29–36 cycles, the PCR products were analyzed in 10% acrylamide gels and stained with ethidium bromide. The amount of the corresponding cytokine mRNA per microgram of total RNA, normalized to the amount of β-actin mRNA, was determined by scanning densitometry and expressed as arbitrary units.

### Intracellular and soluble cytokine production

To assess soluble cytokines, purified CD4^+^ T cells were stimulated by anti-CD3/CD28 mAbs or anti-CD3/*ALL* for 48 h. In addition, other cells were stimulated by 1 µg/mL immobilized anti-CD3 mAb plus 1 µg/mL anti-moesin mAb during 48 h of culture. After culture, 25 μL supernatant was collected and analyzed to measurement of IL-2, IL-4, IL-6, IL-10, IL-17A, IFN-gamma, and TNF by means of a cytometric bead array (CBA) kit following manufacturer's instructions (BD Biosciences). Analyses were obtained by flow cytometry with FCAP Array version 3.0.19.2091 software. The Kit detection limits were as follows: IL-2, 0.1 pg/mL; IL-4, 0.03 pg/mL; IL-6, 1.4 pg/mL; IFN-γ, 0.5 pg/ mL; TNF, 0.9 pg/ mL; IL-17A, 0.8 pg/ mL, and IL-10, 16.8 pg/mL.

For intracellular cytokine detection, cells were activated for 48 h as described and 4 h before brefeldin-A was added (1 μg/μL). At the end of the incubation period, cells were harvested, washed in PBS–BSA buffer, and fixed with 4% *p*-formaldehyde in PBS for 10 min at 4°C. Then, cells were washed twice in PBS and permeabilized with saponine buffer (0.1% saponine, 0.01% pig IgG, 10 mM HEPES, 10% BSA in PBS), shaking gently for 10 min at 4°C. Subsequently, cells were incubated with fluorochrome-labeled Abs against IL-2, IL-4, IL-10, TNF, IFN-gamma, and TGF-beta for 30 min at 4°C. Cells were washed in 0.1% saponine, 100 mM HEPES, 10% BSA in PBS, then washed in PBS–BSA buffer, and analyzed by flow cytometry.

### Isolation of lipid rafts from CD4^+^ T cell activated via CD3/CD28

Purified CD4^+^ T cells were stimulated by optimal concentrations of anti-CD3 mAb plus anti-CD28 mAb for 48 h of culture. Non-stimulated cells were used as control. Once cultured, cells were treated using a slightly modified method 21. In brief, cells (1 × 10^8^) were incubated in 200 μL lysis buffer (1% Triton X-100 in base buffer TEN containing 10 mM Tris-HCl, pH 7.5, 5 mM EDTA, and 150 mM NaCl supplemented with a protease inhibitor mixture from Roche) and shaken for 30 min at 4°C. The cell lysate was sonicated four times × 15 s at 14 Hz frequency with intervals of 30 s on ice and clarified (1500 *g* for 5 min at 4°C). The resulting supernatant was mixed in OptiPrep in base buffer TEN to obtain a solution of 40% OptiPrep; 0.75 mL of the solution were transferred to a 2.5 mL ultra-centrifuge tube (Beckman, Palo Alto, CA). Next, 1.2 mL of 30%, 0.25 mL of 5%-OptiPrep, and 0.25-mL base buffer TEN were sequentially overlaid on top of the clarified cell lysate. The sample was then centrifuged at 60,000 *g* in a TL555 rotor (Beckman) movable angle for 2 h at 4°C. Seven 0.34 mL fractions were collected gradually from the top of the gradient and stored at −70°C. The protein concentration in each OptiPrep gradient fraction was determined by a DC protein assay kit according to manufacturer's instructions (Bio-Rad). BSA was used as the standard.

### Electrophoresis

Equal volumes (30 µg) of the cholesterol-rich membrane protein samples were resolved by 7.5% sodium dodecyl sulfate (SDS)–polyacrylamide gel electrophoresis (PAGE) and stained (0.1% Coomassie blue, 50% methanol, 10% acetic acid). For two-dimensional SDS–PAGE (2-DE), selected fractions (250 μg) were clarified by a ReadyPrep 2D cleanup kit according to the manufacturer's instructions (Bio-Rad). After treatment, the dried pellet was mixed with a rehydration solution (8 M Urea, 2 M thiourea, 0.5% CHAPS, 2% immobilized pH gradient [IPG]-buffer ampholines pH 3–10, 40 mM dithiothreitol [DTT], and 0.002% bromophenol blue) yielding a final volume of 125 μL that was applied to 7 cm IPG strips (Immobiline DryStrip), pH 3–10, and linear gradient in a reswelling tray (all GE Healthcare) and allowed to rehydrate for 18 h. For the first-dimension, IPG strips were resolved by isoelectric focusing using an Ettan IPGphor system (Bio-Rad) according to the described method 22. Afterwards, IPG strips were stored at −70°C until needed. For the second dimension, focused strips were equilibrated for 15 min in 50 mM Tris-HCl, pH 8.8, 6 M Urea, 30% glycerol, 2% SDS, 0.002% bromophenol blue, and 1% DTT, then followed by an identical incubation to replace DTT with 2.5% iodoacetamide. After placing the strips on 7.5% gels, vertical electrophoresis was performed and gels were stained with Coomassie blue solution or transferred to polyvinyldifluoridine (PVDF) Immobilion-P membranes (Millipore Corp, Billerica, MA) or analyzed by Nano liquid-chromatography electrospray-ionization tandem mass spectrometry (LC-ESI-MS/MS) systems.

### Far-Western blotting

For far-Western blotting, resolved proteins in electrophoresis were transferred to PVDF membranes (Millipore Corp. Billerica, MA) as described in previous studies 23,24. In brief, membranes were incubated in a blocking buffer (3% BSA in Tris-buffer-saline-Tween [TBS-Tween] containing 20 mM Tris, pH 7.6, 137 mM NaCl, and 0.1% Tween-20), washed three times (10 min each) in TBS-Tween, incubated at 4°C overnight, and then 2 h at 37°C 23, shaken gently with biotin-*ALL* (400 μg/mL), and followed by 1 h incubation with peroxidase-extravidin (1:5000) or peroxidase-cholera toxin B subunit (1:500). Finally, proteins were visualized by SuperSignal West Pico chemiluminescent substrate (Thermo Scientific, Rockford, IL) on autoradiography film from Kodak Biomax MR (Sigma–Aldrich).

### Nano LC-ESI-MS/MS

Protein spots of interest in 2-DE were identified by comparing proteins stained in Coomassie blue and those recognized through *ALL* in far-Western blotting. Gel spots were excised from the 2-DE with a sterile scalpel. Gel pieces were washed in 50% (v/v) acetonitrile and 25 mM sodium bicarbonate (pH 8.5) for 15 min twice to remove Coomassie dye. Next, samples were dried in a vacuum after dehydration with 100% acetonitrile for 10 min at room temperature, followed by rehydration with sequencing-grade modified trypsin (Promega, Madison, WI) in 25 mM ammonium bicarbonate (pH 8.5) at 37°C overnight. In-gel tryptic digested samples were trapped and desalted isocratically on an LC-Packing PepMap C_18_ µ-pre-column cartridge (Dionex, Sunnyvale, CA) and loaded into an integrated nano-LC-ESI-MS/MS system by an analytical C_18_ capillary column connected online to a quadrupole acceleration time-of-flight, Ultima API, and mass spectrometer (Micromass, Manchester, UK). Instrumental operation, data acquisition, and analysis were carried out under the full control of Mass-Lynx 4.0 (Micromass). The 1-s survey scans were run over in the *m/z* mass range of 400–2000. A maximum of three concurrent MS/MS acquisitions were triggered for 2+, 3+, and 4+ charged precursor detection at an intensity above the predefined threshold. Product ions were analyzed by the Mascot software (www.matrixscience.com) using both NCBInr and EST databases. Parameters for the Mascot search were peptide mass tolerance of 1 Da, MS/MS ion mass tolerance of 1 Da, maximally one missed cleavage and tryptic digestion. Variable modifications included cysteine carbamidomethylation and methionine oxidation 25. Only proteins with ion scores >30 were reported.

### Confocal microscopy

Purified CD4^+^ T cells (2 × 10^6^) were cultured in chamber slides (BD Falcon, San Diego, CA) and activated by anti-CD3 plus anti-CD28 mAbs for 48 h. After removing the supernatant, cells with or without treatment in 1% *p*-formaldehyde (for 5 min), were washed twice in PBS. Cells were incubated with 1% BSA in PBS for 20 min, washed in PBS, and incubated again either with biotin-*ALL* (15 μg/mL) for 2 h, followed by a second incubation with FITC-streptavidin at dilution 1:200, or with rabbit anti-moesin FERM domain mAb, followed by an Alexa Fluor 546-labeled donkey anti-rabbit IgG secondary antibody for 30 min at 4°C in humidity chamber. Cells were then washed in PBS and slides were mounted in Vectashield (containing DAPI dye) at dilution 1:3 in PBS. Cells incubated with FITC-streptavidin or Alexa Fluor 546-secondary antibody were used as controls. For colocalization assays, cells were fixed in 1% *p*-formaldehyde and visualized by microscopy equipped with the ApoTome.2 confocal system (Carl Zeiss, Oberkochen, Germany) using a 63x/1.4 objective lens. The digital images were processed by AxioVision based on Release 4.8.2 SP1 software (Carl Zeiss, Jena, Germany). The images were taken under the same exposure, magnification, and intensification; the processing was the same for all the images shown.

### Statistical analysis

Data were analyzed by Graphpad Prism 5 software (La Jolla, CA) and Origin 8.5.1 sr2 software (Northampton, UK), using a Shapiro–Wilk test to reveal population distributions. Student's *t*-test was performed for comparison of variables that were symmetrically distributed, and values are shown as mean ± standard deviation (SD). To compare groups, an analysis of variance test followed by Bonferron's multiple comparison test was carried out. Values were considered statistically significant at *P *< 0.05.

## Results

### *ALL* enhanced proliferation of CD4^+^ T cells activated by anti-CD3 antibody

**Figure 1:**
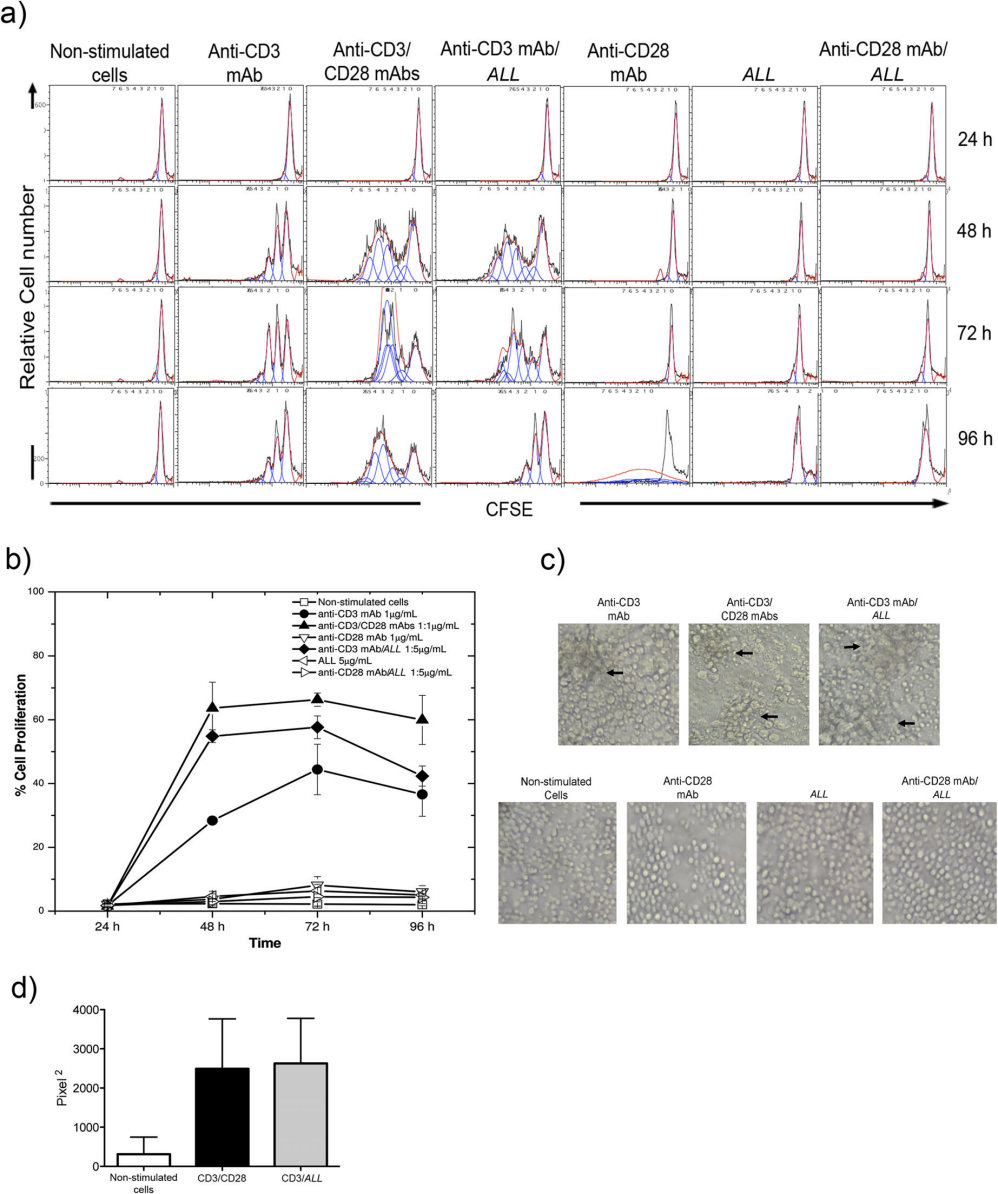
The CD4^+^ T cell proliferation percentage induced by CD3/A. leucocarpus lectin is comparable to that induced by CD3/CD28. Murine lymphoid node CD4^+^ T cells were purified, cultured, and analyzed by flow cytometry and microscopy. (a) Histograms representing cells stained by carboxyfluorescein succimidyl ester (CFSE) and cultured at indicated times. Cell proliferation (sequential halving of fluorescence intensity) was induced under indicated conditions using 1 μg/mL anti-CD3 mAb and either 1 μg/mL anti-CD28 mAb, or 5 μg/mL *ALL*. (b) Graphic showing the cell proliferation percentage on different days of culture under the conditions indicated. (c) Representative photographs showing massive cell clusters shaped via anti-CD3 mAb alone (upper left panel) or in combination with either anti-CD28 mAb or *ALL* (upper middle and right panels) during 48 h of culture. Non-stimulated cells, and cells cultured with anti-CD28 mAb or *ALL* or both *ALL* and anti-CD28 mAb were used as controls (lower panels). Typical clusters of activated cells are pointed out by arrows. Magnification ×20. (d) Measurement in pixel^2^ from area occupied by activated cell clusters. Results are presented as the mean ± standard deviation from three independent experiments.

To confirm whether *ALL* can act as a costimulatory molecule, purified CD4^+^ T cells were simultaneously stimulated via immobilized anti-CD3 mAb plus soluble *ALL*. The results showed 39.5 ± 0.6% CD4^+^ T cell proliferation at 1 μg/mL anti-CD3 mAb, which increased 1.7-fold (69.1 ± 0.9%) in the presence of 5 μg/mL *ALL* (*P* = 0.03) only at 48 h of cell culture. However, under these conditions, at 10, 15, or 20 μg/mL *ALL* did not show the highest increase in cell proliferation. Likewise, *ALL* alone was unable to induce CD4^+^ T cell proliferation, showing (even at high doses) behavior similar to that of non-stimulated cells (data not shown). To compare the effect of *ALL* on CD3-substimulated CD4^+^ T cell proliferation versus that mediated by the CD28 molecule, cells were treated as above, at different times of culture. In Figure 1a, the analysis by histograms showed that CD4^+^ T cell proliferation percentage induced via CD3 plus *ALL* (54.8 ± 2.0%) was comparable to that induced by CD3 plus CD28 at 48 h of culture (63.6 ± 8.1%). The effect of *ALL* on CD3-substimulated CD4^+^ T cells was maintained until 72 h, whereas costimulation by CD28 persisted until 96 h of culture (Fig. 1b). On the contrary, no difference was detected at 24 h of cell culture under any conditions tested (Fig. 1a). The quantitative analysis showed that proliferation index was 1.1-fold higher in cells activated by anti-CD3/CD28 than those cells activated by anti-CD3/ALL at 48 h of culture (Table1). In both cell stimulations, similar results were observed through the division index at 48 h; and the values were the same for cell proliferation index and division index at 72 h of cell activation (Table1). Analysis by microscopy showed typical cell clusters formed through sub-stimulation with immobilized anti-CD3 mAb (1 μg/mL) alone on purified CD4^+^ T cells at 48 h of culture (Fig. 1c). The massive cell clusters increased in size and number in culture of CD4^+^ T cells stimulated via CD3/CD28, which were similar shape in those cells stimulated via CD3/*ALL* (Fig. 1c, upper panels). The quantitative measurement on area occupied by cell clusters showed a same average value between cells stimulated by CD3/CD28 and CD3/*ALL* (Fig. 1d). In contrast, cells incubated with *ALL* or anti-CD28 mAb or *ALL* plus anti-CD28 mAb exhibited a similar shape than non-stimulated cells (Fig. 1c, lower panels).

**Table 1:** Comparative analysis on the cell proliferation of non-stimulated or stimulated CD4^+^ T cells upon indicated conditions

		Non-stimulated cells	Anti-CD3 mAb	Anti-CD3/CD28 mAbs	Anti-CD3 mAb/*ALL*	Anti-CD28 mAb	*ALL*	Anti-CD28 mAb/*ALL*
24 h	Div. index	0.06 ± 0.01	0.05 ± 0.003	0.05 ± 0.001	0.05 ± 0.03	0.04 ± 0.02	0.04 ± 0.05	0.04 ± 0.02
	Prol. index	1.18 ± 0.02	1.06 ± 0.03	1.09 ± 0.007	1.2 ± 0.14	1.09 ± 0.01	1.19 ± 0.15	1.33 ± 0.27
48 h	Div. index	0.06 ± 0.006	0.58 ± 0.02	0.87 ± 0.04	0.66 ± 0.04	0.06 ± 0.008	0.15 ± 0.05	0.089 ± 0.003
	Prol. index	1.18 ± 0.02	1.3 ± 0.17	2.12 ± 0.17	1.83 ± 0.09	1.36 ± 0.06	1.23 ± 0.14	1.1 ± 0.04
72 h	Div. index	0.07 ± 0.005	0.39 ± 0.02	0.59 ± 0.03	0.47 ± 0.08	0.18 ± 0.02	0.076 ± 0.009	0.065 ± 0.01
	Prol. index	1.19 ± 0.04	1.17 ± 0.09	2.33 ± 0.16	2.32 ± 0.11	1.9 ± 0.10	1.24 ± 0.10	1.16 ± 0.10
96 h	Div. index	0.08 ± 0.008	0.385 ± 0.03	0.77 ± 0.08	0.32 ± 0.04	0.605 ± 0.03	2.76 ± 0.79	0.016 ± 0.005
	Prol. index	1.18 ± 0.02	1.19 ± 0.04	2.31 ± 0.15	1.21 ± 0.02	1.15 ± 0.04	1.92 ± 0.26	2.56 ± 0.42

Division Index (Div. Index) is the average number of divisions for all cells in the culture. Proliferation Index (Prol. Index) is the measurement for responding cells, denotes the average number of divisions that cells have undergone.

### CD4^+^ T cells stimulated by anti-CD3 mAb plus *ALL* produce cytokines

**Figure 2:**
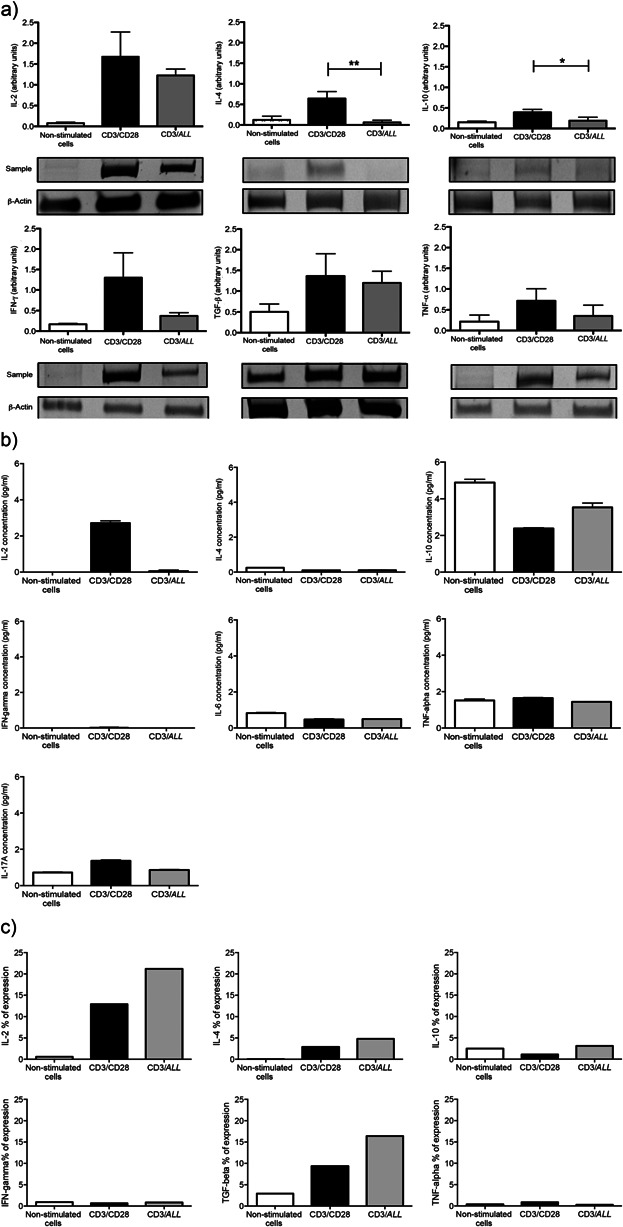
The production of cytokines in CD4^+^ T cells induced by CD3/*A. leucocarpus* lectin as compared to that induced by CD3/CD28. Murine lymph node CD4^+^ T cells were stimulated via CD3/CD28 or CD3/*ALL* for 48 h. (a) Products from PCR assay for indicated cytokine transcripts were resolved by 10% PAGE–SDS. The amount of cytokine mRNA per microgram of total RNA was normalized to the amount of β-actin mRNA by scanning densitometry. Bars represent mean ± standard deviation of three independent experiments, which are expressed as arbitrary densitometry units. (b) Supernatant from cell cultures was collected and analyzed by CBA kit for measurement of indicated soluble cytokines. Bars represent mean ± standard deviation of three independent experiments. (c) Intracellular cytokines in cells stimulated for 48 h. Bars denote mean of two independent experiments.

To evaluate cell function, CD4^+^ T cells were simultaneously stimulated by anti-CD3 mAb plus *ALL*. The results showed similar levels of transcripts for IL-2, TNF-α, TGF-β, and IFN-γ compared to those shown by CD3/CD28-stimulated CD4^+^ T cells (Fig. 2a). However, cells activated via CD3/*ALL* showed a lower production of mRNA for IL-4 and IL-10 than those cells activated by CD3/CD28. The difference was statistically significant for both IL-4 (0.06 ± 0.05 vs. 0.64 ± 0.19; *P* = 0.005, respectively) and IL-10 (0.19 ± 0.08 vs. 0.39 ± 0.07; *P* = 0.03, respectively) (Fig. 2a). With regard to the levels of soluble cytokines measured in cell culture supernatant, the IL-2 was 67-fold higher in cells activated by CD3/CD28 than in those cells activated by CD3/*ALL* (*P* < 0.005). On the order hand, IL-10 was 1.5-fold higher in the culture supernatant of cells activated by CD3/*ALL* than in cells activated by CD3/CD28 (*P* < 0.05) as shown in Figure 2b. Other cytokines did not show significant differences in their amounts, the levels of both IFN-γ and IL-4 were almost undetectable (Fig. 2b). Concerning to the measurement of intracellular cytokines, there were a higher percentage of cells positive to IL-2 and TGF-β in the culture of cells activated by CD3/*ALL* than in cells activated by CD3/CD28 (Fig. 2c).

### *A. leucocarpus* lectin recognizes a receptor associated to lipid rafts

**Figure 3:**
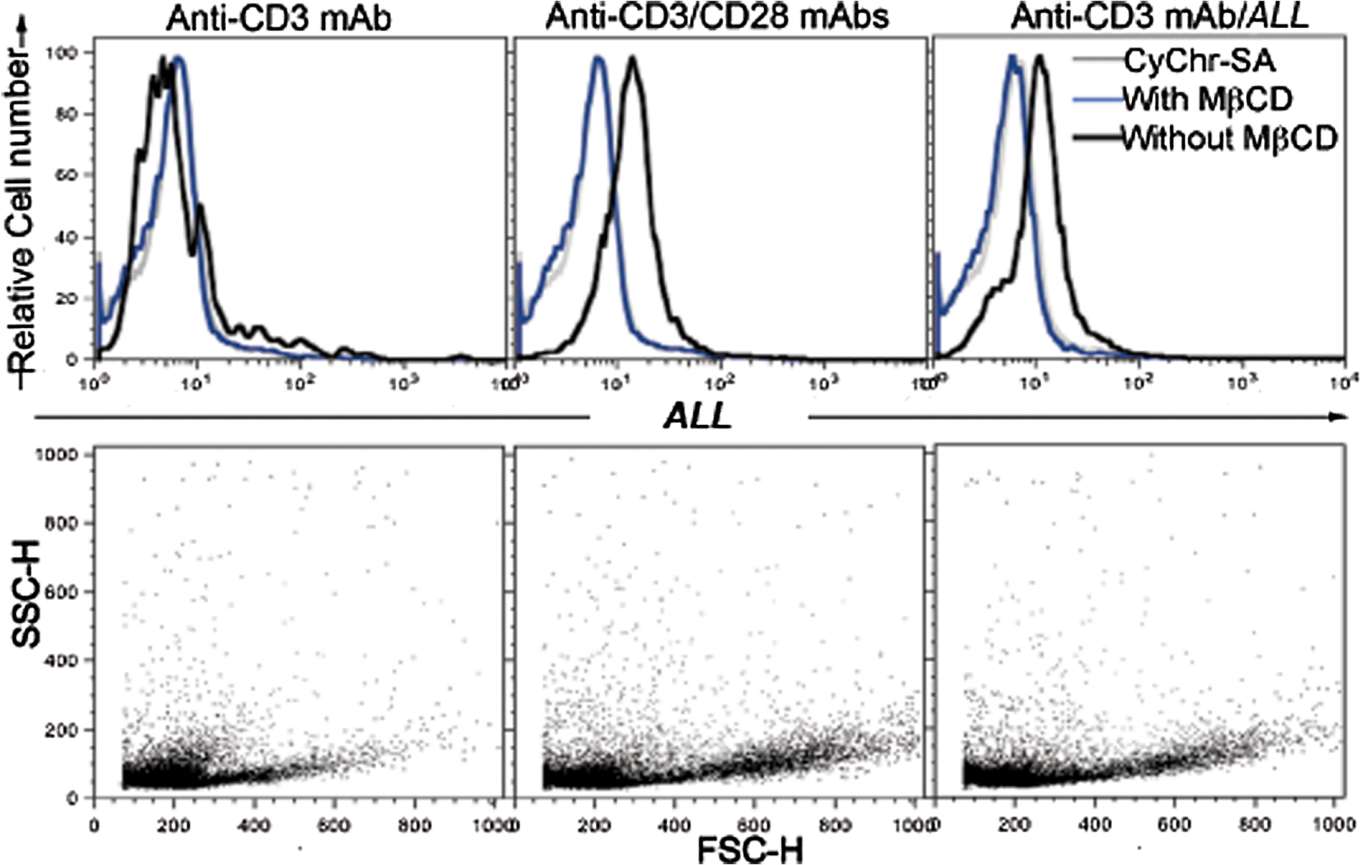
Glycoprotein recognized by *A. leucocarpus* lectin is associated to lipid rafts. Murine lymph node CD4^+^ T cells were cultured during 48 h. Representative histograms of fluorescent cells and dot plots of proliferating cells analyzed by flow cytometry. Cells were activated via anti-CD3 mAb alone (left panels) or in the presence of anti-CD28 mAb or *ALL* (middle and right panels). After culture, cells were treated (blue line in histograms) or not (black line in histograms) with methyl-β-cyclodextrin (MβCD). Next, cells were incubated with biotin-*ALL* followed by the CyChr-streptavidin (CyChr-SA) fluorescent staining. CyChr-SA as staining control (gray line in histograms) was used. Results are representative of three independent experiments.

Because freshly obtained mouse CD4^+^ T cells incubated with FITC-labeled *ALL* showed formation of fluorescent patching and capping on cell surface (data not shown), we examine whether cholesterol depletion in lipid rafts affects cell membrane recognition by *ALL*. For this, CD4^+^ T cells were treated with MβCD after activation via CD3/CD28 or CD3/*ALL* for 48 h. Results showed that cells sub-stimulated by immobilized anti-CD3 antibody alone at a dose of 1 μg/mL, a low cell proliferation was observed (Fig. 3, lower left panel). Under this condition, *ALL* recognized 27% of the cells, which was lost after treatment with MβCD (Fig. 3, upper left panel). In contrast, cells stimulated by anti-CD3 plus anti-CD28 mAbs, the percentage of *ALL*-positive cells increased 2.8-fold (Fig. 3, upper middle panel). Similarly, cells stimulated by anti-CD3 mAb plus *ALL*, the percentage of cells positive to *ALL* increased 2.3-fold (Fig. 3, upper right panel). Furthermore, the recognition of proliferating cells by *ALL* was returned to a background level after treatment with MβCD (Fig. 3, upper middle and right panels).

### *A. leucocarpus* lectin recognizes two prominent proteins from lipid rafts

**Figure 4:**
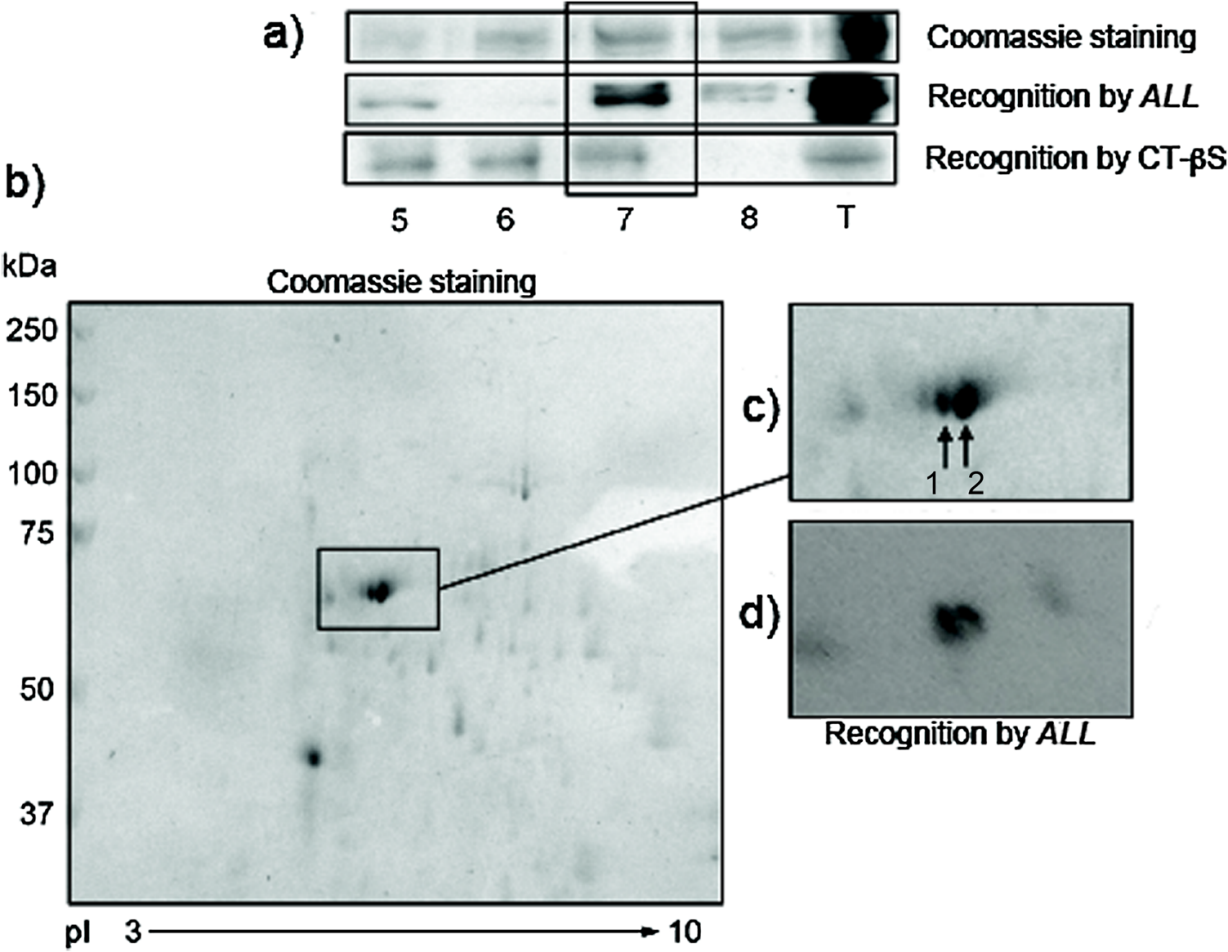
*A. leucocarpus* lectin recognizes two prominent proteins from lipid rafts. Murine lymph node CD4^+^ T cells were stimulated by CD3/CD28 during 48 h. (a) Protein bands close to 70 kDa were detected in 7.5% SDS–PAGE and far-Western blotting in isolated cholesterol-rich membrane fractions (lanes 5–8) and total lysed sample (lane T). Gel was visualized by staining with Coomassie blue (upper panel). By far-Western blotting, *ALL* recognized two protein bands (lanes 5, 7–8 and T; middle panel), whereas cholera toxin β subunit (CT-βS) reacted to 5–7 fractions and total lysed sample (lanes 5–7 and T; lower panel). (b) The 2-DE stained by Coomassie blue shows two close prominent protein spots, (c) which are indicated by numbered arrows on amplified mode. (d) The same protein spots were also recognized by *ALL*. Molecular weight markers are indicated alongside the gel. Their identities are listed in Table1. The reactivity was visualized by chemiluminescence. Results are representative of six independent experiments.

The far-Western blotting analysis of the resulting lipid raft fractions (isolated from CD3/CD28-activated CD4^+^ T cells during 48 h) showed a marked reactivity of *ALL* on two bands from fraction 7 (Fig. 4a, middle panel), which were also recognized by the cholera toxin B subunit (Fig. 4a, lower panel). Additionally, *ALL* recognized bands in fractions 5 and 8 but at a lesser extent (Fig. 4a, middle panel). Here, the cholera toxin B subunit was used as a reagent to confirm the isolation of lipid rafts. Subsequently, to identify the cholesterol-rich membrane proteins recognized by *ALL*, a far-Western blotting of fractions 5 and 7 was again performed after 2-DE. In fraction 7, around 40 protein spots were observed in Coomassie staining (Fig. 4b), where two prominent protein spots with mobility relatively close to 70 kDa were selected (amplified region in Fig. 4c). The selected protein spots in turn were recognized by *ALL* (Fig. 4d). Same results were obtained from fraction 5, but at a lower intensity than from fraction 7 (data not shown). The *ALL*-recognized protein spots resulting from fraction 7 were subjected to mass spectrometry and compared with the protein database. The analyzed protein spots are shown in Table2. The number one exhibited a 33% homology to matricin (gi|347839 NCBInr) with a theoretical mass of 61 kDa, whereas the number two showed 41% identity to an unnamed protein closely related to moesin, with a theoretical mass of 67.8 kDa (gi|74186081 NCBInr) and 6.3 pI. This last protein sequence was also analyzed by both the protein structure prediction system, Phyre^2^ 26 and NetOGlyc v 4.0 software 27. The Phyre^2^ system showed 90% identity to the FERM domain of moesin, whereas the second showed nine potential O-glycosylation sites; which the positions 165, 205, and 206 exhibited 0.68, 0.78, and 0.86 scores, respectively.

**Table 2:** *A. leucocarpus* lectin far-WB-bound proteins from activated CD4^+^ T cells identified by LC Ms/Ms

NCBInr accession	Spot No*	Protein	Theoretical mass (kDa)	Peptides matched	Sequence coverage**
gi|347839	1	Matricin	61	13	33%
gi|74186081	2	Unnamed protein product	67.8	15	41%

Only proteins that showed significant ion scores (>33) are indicated.

^*^ALL-positive spots identified in Figure 3e.

^*^^*^Represents the identity or extensive homology (*P* < 0.05) with the indicated protein.

### Staining by *ALL* colocalizes with anti-moesin FERM domain antibody in activated CD4^+^ T cell surface

**Figure 5:**
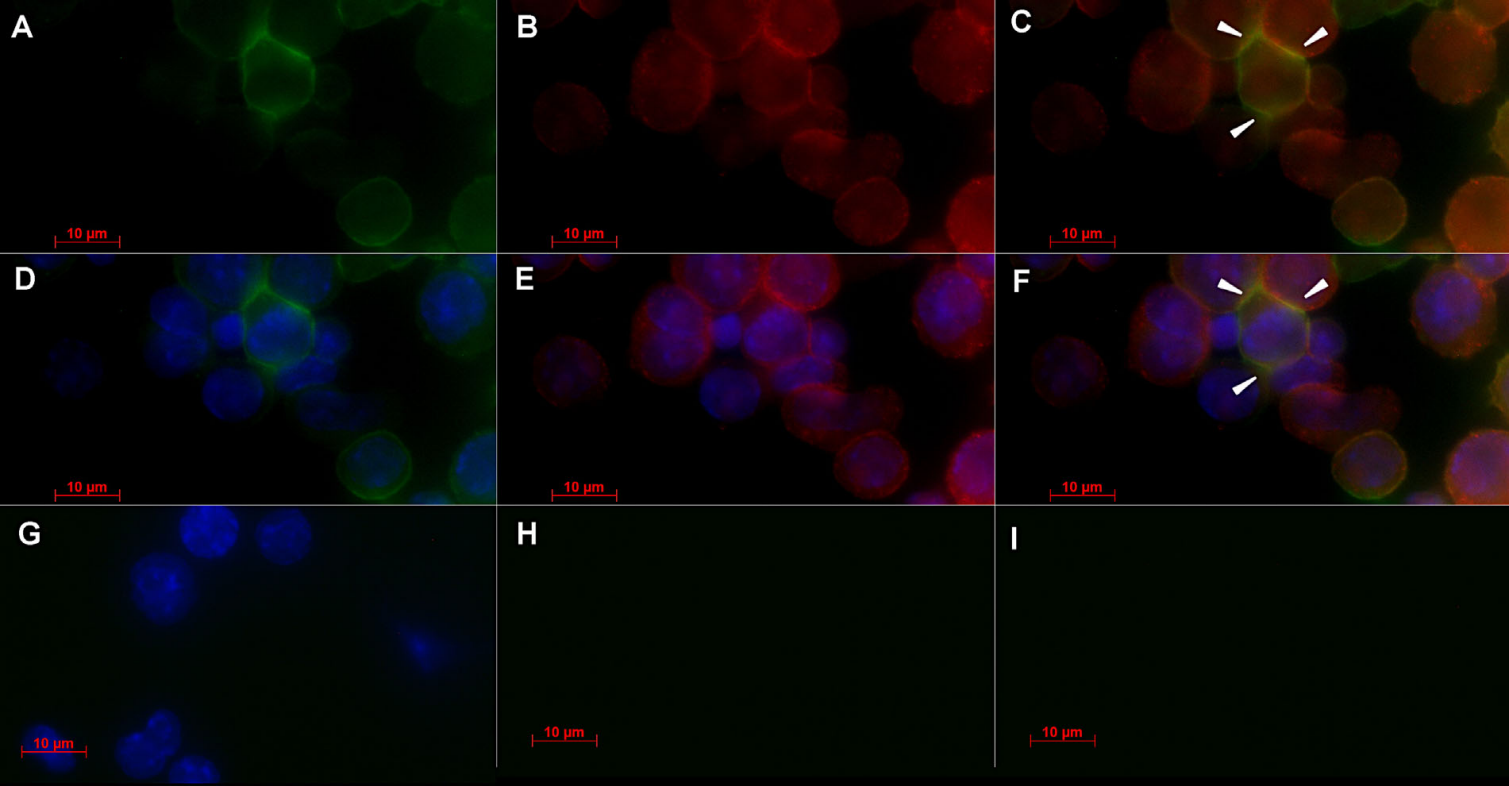
Glycoprotein recognized by *A. leucocarpus* lectin colocalizes with protein recognized by anti-moesin FERM domain antibody in activated CD4^+^ T cell surface. Murine lymph node CD4^+^ T cells were activated via CD3/CD28 for 48 h of culture. Then, non-permeabilized cells were fixed and stained by triple immunofluorescence. The images are part of a reconstructed Z series, and the individual sections were taken along the *x–y* axes. (a) Image from cells stained with biotin-*ALL* followed by FITC-streptavidin. (b) Image from cells stained by rabbit anti-moesin FERM domain mAb and followed by an Alexa Fluor 546-donkey anti-rabbit IgG secondary antibody. (c) Merged image from cells stained by *ALL* (green) and anti-moesin mAb (red). (d–f) DAPI nuclear staining (blue) was used as a cellular counterstain. (g) Cells stained with DAPI only as control. (h–i) Cells incubated with FITC-streptavidin or Alexa 546-secondary antibody were used as staining control. Images were visualized by microscopy equipped with the ApoTome.2 confocal system (Carl Zeiss). Data shown are representative of three individual experiments. Arrowheads indicate recognition by *ALL* and anti-moesin mAb. Bars 10 µm.

To test whether *ALL* recognizes a FERM family-bearing protein, CD4^+^ T cells were activated via CD3/CD28 for 48 h, after, stained with both *ALL* and anti-moesin FERM domain mAb. Staining was performed on undamaged cells, since they had not been treated with permeabilizing reagents. The fluorescent staining by *ALL* was observed along of cell surface only in *ALL*-positive cells, which seem be concentrated at intercellular contact sites (Fig. 5A, D). Interestingly, the vast majority of cells were recognized by anti-moesin mAb, and in some cells, the fluorescent staining was also intense at cellular interaction sites (Fig. 5B, E). Cells incubated with secondary reagents did not showed fluorescent staining (Fig. 5G–I). The colocalization events were observed at intercellular contact sites in the agglutinated *ALL*-positive cells (Fig. 5C, F).

### CD4^+^ T cells activated by anti-CD3/anti-moesin mAbs secrete cytokines

To examine whether the anti-moesin FERM domain mAb costimulates CD3-substimulated CD4^+^ T cells, purified CD4^+^ T cells were simultaneously activated by anti-CD3 mAb plus anti-moesin mAb for 48 h. Results showed activated cell clusters similar to those from cells stimulated by CD3/CD28 or CD3/*ALL*. The measurement in pixels^2^ was 312.9 ± 435.8 to non-stimulated cells; 2492 ± 1275 to cells upon stimulation by CD3/CD28; 2629 ± 1150 to CD3/*ALL*; and 2393 ± 1246 to CD3/moesin. Likewise, level of cytokines in culture supernatant from cells activated by CD3/moesin was very similar to those cells activated via anti-CD3 mAb plus *ALL* (Table3). Nevertheless, the concentration of IL-17A in CD3/*ALL*-activated cells was higher than CD3/moesin-activated cells (Table3).

**Table 3:** Soluble cytokines detected in culture supernatant of non-stimulated or stimulated CD4^+^ T cells upon indicated conditions at 48 h of culture

	Non-stimulated cells	CD3/CD28	CD3/*ALL*	CD3/Moesin
IL-10	4.8 ± 0.17	2.3 ± 0.03	3.5 ± 0.2*	2.4 ± 0.02*
IL-17A	0.7 ± 0.02	1.3 ± 0.04	0.86 ± 0.02	0.01 ± 0.01
TNF-α	3.0 ± 0.07	1.6 ± 0.02	1.4 ± 0.01	1.3 ± 0.007
IL-6	0.8 ± 0.03	0.4 ± 0.03	0.4 ± 0.01	0.5 ± 0.02
IL-2	0 ± 0	2.71 ± 0.12**	0.04 ± 0.06**	0.03 ± 0.04**
IL-4	0.2 ± 0.007	0.1 ± 0.01	0.1 ± 0.01	0.07 ± 0.01
IFN-γ	0 ± 0	0.02 ± 0.02	0 ± 0	0 ± 0

Mean ± Standard deviation. Results are in pg/mL. For kit detection limits, see experimental section.

^*^P < 0.05.

^*^^*^P < 0.005.

## Discussion

The role of glycans to induce T cell activation can be shown by the use of lectins, which are proteins or glycoproteins with reversible binding to their saccharide ligands 28. The lectins have at least one non-catalytic domain that recognizes oligosaccharides in a specific spatial conformation 28. One of these lectins, *ALL* with specificity for GalNAc in either the Thomsen–Friedenreich antigen (Galβ1,3GalNAc α1,O-Ser/Thr) or Tn antigen (GalNAc α1,O-Ser/Thr) 8 was used in this work. The role of *ALL* in T cell costimulation has been reported 15; however, we showed that in the absence of APCs, *ALL* enhanced CD4^+^ T cell proliferation when given a CD3-based stimulation, which was comparable to stimulation via CD3/CD28. The CD28 molecule is known as the major T cell costimulatory receptor for the production of IL-2 29. Thus, the production of cytokines was determined in *ALL*/CD3-stimulated cells; in this sense, non-CD28 T cell costimulatory molecules have been found to induce cytokines production. Among these molecules, the stromal cell-derived factor-1 α costimulates cell proliferation and production of IL-2, IL-4, IL-10, and IFN-γ on activated CD4^+^ T cells 30. Monoclonal antibodies to CD5 or CD9 induce also potent [^3^H]TdR uptake and IL-2 production on activated T cells 31. Likewise, concurrent CD2 and CD48 costimulation enhances CD3-driven T cell activation concerning both proliferation and synthesis of transcript for IL-2 and IFN-γ 32. Nevertheless, T cell costimulation by CD5, CD9, or CD2-CD48 interaction is less efficient than CD28 31,32. In our case, the production of transcripts for IL-2, TNF- α, TGF-β, and IFN-γ in CD4^+^ T cells activated via CD3/*ALL* did not show statistical differences comparing with those activated via CD3/CD28. Concerning to secreted cytokines, we observed that IL-2 is more abundant in the culture of cells activated through CD3/CD28 in comparison to the other conditions of culture, possibly by positive feedback during the activation 1. Although the costimulation via *ALL* appears to be insufficient for production of IL-2, the number of cells positive for IL-2 and TGF-β were higher than the costimulation by CD3/CD28. Altogether our results suggest that the major *ALL*-recognized glycoprotein is able to costimulate the cytokine production, reflecting the T cell functional activity.

On the basis of that costimulatory molecules are found in lipid raft during T cell activation and because T cells incubated with *ALL* display formation of patching and capping on cell surface, we used MβCD to deplete cholesterol from lipid raft after activation of CD4^+^ T cells. Previous studies of our laboratory have been shown that *ALL* binds 22% non-stimulated CD4^+^ T cells, and the number of *ALL*-positive cells increases three-fold after 24–48 h activation 13. The disruption of lipid rafts by treatment with MβCD caused loss in *ALL* recognition in cells activated by 48 h, suggesting that *ALL* binds to glycoproteins recruited or located in lipid rafts from activated CD4^+^ T cells. The membrane raft microdomains are known as the signaling complexes that process intracellular reaction cascades, where diverse molecules participate in signal transduction 1. We selected two prominent protein spots with molecular mass close to 70 kDa from lipid raft membranes resolved by two-dimensional electrophoresis. The two protein spots were recognized by *ALL*, one of them showed the highest score of identity (41%) with an unnamed protein (gi|74186081) closely related to moesin. This protein sequence was also analyzed by NetOGlyc 4.0 software 27 showing nine potential sites for O-glycosylation, of which four of them exhibited a very high probability of being recognized by *ALL*. Previous studies have shown that the binding site of *ALL* interacts with inner core of the Thomsen–Friedenreich antigen, and that *ALL* binds GalNAc residues, which are separated by four amino acids, whereas GalNAc residue clusters prevent recognition by *ALL* 33. Another analysis on protein sequence was performed using Phyre^2^ 26 that showed 90% identity with the FERM domain of moesin. The FERM domain defines members of the band 4.1 family, which includes cytoskeletal proteins, such as the ezrin–radixin–moesin (ERM) protein family, as well as several tyrosine kinases and phosphatases 34. The ERM proteins play an important role in the interface between plasma membrane proteins and major cytoskeletal structures that regulate membrane-associated signalling pathways, as reviewed 4. Data from confocal immunofluorescence staining showed that *ALL* and anti-moesin FERM domain mAb colocalized in cellular contact sites, indicating the presence the O-glycans recognized by *ALL* in these zones. Changes in glycosylation of cell surface O-glycoproteins during T cell activation have been reviewed 2,35. The decrease of acid sialic on activated T cells 36, allows exposition of GalNAc or Galβ1,3GalNAc moieties on cell surface glycoproteins, which could be recognized by *ALL*. However, as observed in immunofluorescence image, some agglutinated cells were strongly stained by *ALL* and weakly bound with anti-moesin mAb and viceversa. Moreover, other agglutinated cells were not recognized either by *ALL* or anti-moesin mAb, suggesting that not all moesin-like proteins display the same glycosylation profile and that different O-glycoproteins could be recognized by *ALL*. Diverse studies have evidenced the importance of O-glycans on cell surface because they seem be involved in activation process of the T cell. Comelli E.M. et al. reported that the activation of murine T cells lead to a marked decrease of N-glycans carrying NeuAcα2,6Gal and the increase of O-glycans carrying the disaccharide Galβ1,3GalNAc on the cell surface 37. Similarly, van Vliet et al. showed that the activation of human T cells via CD3/CD28 was accompanied by an increase in the expression of Galβ1,3GalNAc on the cell surface 38. We reported in this study direct visualization of a moesin-like protein on the activated CD4^+^ T cell surface. Although moesin is localized in the sub-membranous cytoskeleton, some authors have reported moesin-like proteins on the leukocyte surface 39,40. Ariel A. et al. demonstrated that a moesin-like protein is expressed on a membrane of human resting T cells, which significantly increased on cell surface after 48 h activation 41. Interestingly, the anti-moesin FERM domain antibody was able to enhance CD3-based stimulation in CD4^+^ T cells after 48 h of culture, being the first time that an anti-moesin FERM domain antibody displays a costimulatory function.

## Conclusions

Our findings pointed out that the major glycoprotein recognized by *ALL* was able to costimulate the production of cytokines; *A. leucocarpus* lectin binds two glycoproteins situated in lipid raft microdomains from activated CD4^+^ T cells; one of which showed identity to a moesin-like protein that was visualised on activated-CD4^+^ T cell surface; and that anti-moesin antibody was able to costimulate CD4^+^ T cells activated by CD3.
